# Antioxidant Activity of Grapevine Leaf Extracts against Oxidative Stress Induced by Carbon Tetrachloride in Cerebral Cortex, Hippocampus and Cerebellum of Rats

**DOI:** 10.3390/antiox3020200

**Published:** 2014-04-02

**Authors:** Mariane Wohlenberg, Daniela Almeida, Liane Bokowski, Niara Medeiros, Fabiana Agostini, Cláudia Funchal, Caroline Dani

**Affiliations:** 1 Centro Universitário Metodista IPA, Coronel Joaquim Pedro Salgado 80, Porto Alegre 90420-060, Brazil; E-Mails: marianefw@yahoo.com.br (M.W.); danielacamposdealmeida@gmail.com (D.A.); lianevvb@gmail.com (L.B.); niarasm@yahoo.com.br (N.M.); csfunchal@yahoo.com.br (C.F.); 2 Universidade de Caxias do Sul, Francisco Getúlio Vargas 1130, Caxias do Sul 95070-560, Brazil; E-Mail: mestrefabi@yahoo.com.br

**Keywords:** grapes, *Vitis labrusca*, total polyphenols, neuroprotection

## Abstract

In recent years, it has become increasingly important to study the beneficial properties of derivatives of grapes and grapevine. The objective of this study was to determine the antioxidant activity of *Vitis labrusca* leaf extracts, comparing conventional and organic grapevines, in different brain areas of rats. We used male Wistar rats treated with grapevine leaf extracts for a period of 14 days, and on the 15th day, we administered in half of the rats, mineral oil and the other half, carbon tetrachloride (CCl_4_). The animals were euthanized by decapitation and the cerebral cortex, hippocampus and cerebellum were removed to assess oxidative stress parameters and the activity of antioxidant enzymes. Lipid peroxidation levels (TBARS) were unchanged. However, CCl_4_ induced oxidative damage to proteins in all tissues studied, and this injury was prevented by both extracts. Superoxide dismutase (SOD) activity was increased by CCl_4_ in the cerebral cortex and decreased in other tissues. However, CCl_4_ increased catalase (CAT) activity in the cerebellum and decreased it in the cerebral cortex. The SOD/CAT ratio was restored in the cerebellum by both extracts and only in the cerebral cortex by the organic extract.

## 1. Introduction

*Vitis labrusca* is the main species used in the manufacture of juices and wines in South America [1]. There are various ways to grow grapes, which include conventional and organic cultivation. In organic cultivation, chemicals that interfere with productivity are not employed, unlike in conventional cultivation, where pesticides are used to control pests [2]. Currently, it is known that grape juices produced from organic cultivation contain higher amounts of polyphenols when compared to grapes from conventional cultivation [1].

Polyphenols are present in grapes and their derivatives [1,3,4]. These bioactive compounds are synthesized by plants and stored in the skin in response to fungal attack, mechanical damage and ultraviolet rays. Some human health benefits have been attributed to polyphenols in recent years, including their antioxidant action on reactive species [5,6]. These phenolic compounds, e.g., catechin, resveratrol, flavonoids, quercetin, rutin, kaempherol and naringin, were found in the leaves of *V. labrusca* grapevines in previous studies [7,8].

Reactive species are molecules derived from various biological processes capable of causing DNA, protein and lipid damage [9]. This damage generated by reactive species is combated by antioxidant defenses, enzymatic and non-enzymatic. Antioxidant enzymes include superoxide dismutase (SOD) and catalase (CAT), while α-tocopherol (vitamin E), β-carotene (pro-vitamin A) and ascorbic acid (vitamin C) are some of the non-enzymatic antioxidants [10]. In normal situations, the human body produces antioxidants or procures them from the diet, and either way, they are capable of reducing the concentration of reactive species [11]. The imbalance between the production of reactive species and amount of antioxidants characterizes oxidative stress [12]. Damage caused by oxidative stress to the central nervous system has been associated with the development of neurodegenerative diseases such as Parkinson’s disease and Alzheimer’s disease [9,13].

Some studies, *in vivo* and *in vitro*, have shown that products derived from grapes or grape leaves, seeds or juices are able to inhibit the damage caused by oxidative stress in the central nervous system [8,14]. Two studies demonstrated the beneficial properties of these derivatives (flavan-3-ol compounds and grape juice) on oxidative damage, in this case induced by pentylenetetrazol (PTZ) [15,16]. An *in vitro* study showed that extracts of leaves from organic and conventional grapevines were able to exert protective effects against the oxidative damage (lipid peroxidation and protein oxidation) and enzymatic (SOD and CAT) changes in liver, kidney and heart caused by hydrogen peroxide (H_2_O_2_) treatment [7]. There are numerous substances capable of inducing damage by oxidative stress, such as carbon tetrachloride (CCl_4_) [17,18]. CCl_4_ is toxic partly because of its liposolubility, and when bound to proteins and lipids, it induces tissue degeneration, which may lead to hepatic steatosis, tubular necrosis and hepatic encephalopathy [17,18,19].

Only *in vitro* studies have demonstrated the neuroprotective activity of *V. labrusca* leaf extract, and no *in vivo* investigations have been reported confirming this activity. The present study aimed to evaluate the reduction of oxidative stress parameters in cerebral cortex, hippocampus and cerebellum of male Wistar rats after chronic treatment with the *V. labrusca* leaf extracts, comparing organic and conventional grapevines.

## 2. Experimental Section

### 2.1. Reagents

Thiobarbituric acid was purchased from Merck (Darmstadt, Germany), and 2,4-dinitrophenylhydrazine (DNPH) was obtained from Sigma (St. Louis, MO, USA). All other chemicals were of analytical grade and purchased from local suppliers.

### 2.2. Grapevine Leaf Extracts

*V. labrusca* leaves, variety Bordo, from both conventional and organic grapevines, were collected in November 2011. The plants were identified by the Herbarium of the Centro Universitario Metodista do IPA. The organic grapevines received the ECOVIDA certification. The leaves were weighed and digested using Xpress digestion tubes (CEM Corporation, Matthews, NC, USA). Afterwards, Milli-Q (Millipore Corporation, Billerica, MA, USA) water was added and to the tubes containing the leaves, and the mixture was subjected to microwave digestion. The samples were then transferred to a flask with Sarstedt focuser (Sarstedt Inc., Newton, NC, USA) with more Milli-Q water added. The samples were centrifuged and the supernatant obtained was used in all experiments.

### 2.3. Phenolic Compounds

The concentration of phenolic compounds was determined using the method described by Singleton and Rossi (1999), a modification of the Folin-Ciocalteau colorimetric method [20]. The results were expressed as mg/100 g leaves.

The extracts were analyzed by high performance liquid chromatography (HPLC) using an 1100 HPLC system (Agilent Technologies, Santa Clara, CA, USA) and the test standards chlorogenic acid, taxifolin, ferrulic acid, naringin, rutin, quercetin, luteolin, kaempferol, apigenin, catechin, epicatechin and resveratrol. The 1100 HPLC system consisted of a quaternary pump system, LiChrospher RP-18 (5 μm) column and UV detector. An injection volume of 50 μL and a flow rate of 1.0 mL/min were used. The quantification of catechin was performed as described by Saucier *et al.* (2001) [21]. Solvent A was water with 5% acetic acid; solvent B was methanol with 5% acetic acid; the elution was isocratic at 90% A and 10% solvent B, with a total analysis time of 45 min and detection at 280 nm. For flavonoid standards, solvent A was methanol with 2% acetic acid and solvent B was Milli-Q water with 2% acetic acid, with a total analysis time of 50 min and detection at 350 nm [22]. For quantification of resveratrol, solvent A was water with pH 2.5 and solvent B, methanol; a gradient of 90% solvent A and 10% solvent B to 100% solvent B was used, a total analysis time of 45 min and detection at 313 nm [23]. All standards were purchased from Fluka Analytical (St. Gallen, Switzerland) or Sigma-Aldrich (St. Louis, MO, USA).

### 2.4. Animals

Thirty male Wistar rats, 90 days old and weighing approximately 300 g, were obtained from the animal facility of the Centro Universitário Metodista IPA. The animals had free access to water and commercial diet containing 20.5% protein (predominantly soybean), 54% carbohydrate, 4% fat, 4.5% fiber, 7% ash and 10% moisture. The animals were maintained on a 12 h light/12 h dark cycle at a temperature of 22 °C ± 1 °C. All experimental procedures were performed with the approval of the Ethics Commission for Animal Use (CEUA) of the Centro Universitário Metodista IPA, No. 01/2012.

### 2.5. Treatment

All animals received once a day a single intraperitoneal injection for 14 days. On the basis of a previous study [24], the animals were divided into three experimental groups (*n* = 10 each). Group 1 (control) received saline (0.9% NaCl); Group 2 received conventional grapevine leaf extract (30 mg/kg); and group 3 received organic extract (30 mg/kg). On the 15th day, half of the animals in each group received a single intraperitoneal dose of CCl_4_ (3 mL/kg) and the other half, a single dose, also intraperitoneally, of mineral oil (control). After 4 h, the animals were euthanized by decapitation. The cerebellum, hippocampus and cerebral cortex were dissected, homogenized in 1.5% KCl with a manual homogenizer, and stored in a freezer (−80 °C) until analysis.

### 2.6. Parameters of Oxidative Stress

As an index of lipid peroxidation, we used thiobarbituric acid reactive species (TBARS) production during an acid-heating reaction, which is widely adopted as a sensitive method for measuring lipid peroxidation, as previously described by Wills (1996) [25]. Briefly, the samples were mixed with 10% trichloroacetic acid (TCA) and 0.67% thiobarbituric acid (TBA) and then heated in a boiling water bath for 15 min in closed tubes. TBARS were determined by absorbance at 535 nm. Results were expressed as nmol/mg protein. Oxidative damage to proteins was assessed by determining carbonyl groups on the basis of the reaction with dinitrophenylhydrazine (DNPH), as previously described by Levine *et al.* (1990) [26]. DNPH reacts with protein carbonyls to form hydrazones that can be measured spectrophotometrically. Briefly, 500 μL 10 mM DNPH in 2 M HCl were added at room temperature for 1 h, with vortexing every 10–15 min. Next, 500 μL 20% TCA were added, and tubes were mixed and centrifuged for 3 min. The supernatant was then discarded, and the pellets were washed three times with 1 mL of ethanol-ethyl acetate (1:1) to remove free reagent. After centrifugation, the precipitated protein was redissolved in 0.6 mL guanidine solution. Proteins were dissolved within 15 min at 37 °C. The insoluble material was removed by centrifugation in a microcentrifuge for 3 min. The absorbance was read at 370 nm. Equal amounts of protein samples without DNPH were used as controls. The results were expressed in nmol/mg protein.

### 2.7. Determination of Antioxidant Enzymes

SOD activity was determined by a spectrophotometric method, measuring the inhibition of the rate of adrenochrome formation at 480 nm (SP-2200 Spectrophotometer, Bioespectro, Curitiba, Brazil) in medium containing 1 mM adrenalin and 50 mM glycine [27]. The results were expressed in U SOD/mg protein. The method used to determine CAT activity has been described by Aebi (1984) [28] and determines the rate of H_2_O_2_ degradation measuring absorbance at 240 nm (Spectrophotometer SP-2200, Bioespectro). The results were expressed as UCAT/mg protein.

### 2.8. Protein Determination

Protein concentration was determined according to the method described by Lowry *et* *al.* (1951) [29].

### 2.9. Statistical Analysis

The results were expressed as the mean and standard deviation. The data were evaluated for normality using the Kolmogorov-Smirnov test. We observed a normal distribution of the data. The differences between all the groups were analyzed by three-way analysis of variance (ANOVA), followed by Tukey’s post-test. Student’s *t*-test was used for comparison between two means. *P* < 0.05 was considered significant. All analyses were performed using the statistical program Statistical Package for the Social Sciences (SPSS) version 17.0 (International Business Machines Corporation, New York, NY, USA).

## 3. Results and Discussion

Our study evaluated the effect of chronic treatment with *V. labrusca* leaf extracts prepared from organic and conventional grapevines, on oxidative stress parameters in cerebral cortex, hippocampus and cerebellum of male Wistar rats. Products from grapevines are known to be some of the most important sources of polyphenols. We found that both extracts are sources of these compounds, including resveratrol and catechin (Table 1). The organic extract was richer in total polyphenols and resveratrol when compared with the conventional extract. However, conventional cultivation favored increased levels of catechin. Our findings are corroborated by several previous studies that have demonstrated that the way grapes are grown influences the amount of total polyphenols [8,24] and concentration of catechin, as shown by Dani *et al.* (2010) [8]. In organic cultivation, pesticides are not used, resulting in a larger amount of polyphenols synthesized by the plant under these conditions [30]. However, another related work comparing organic and conventional grape juice found that organic grape juice was richer in all the phenolic compounds found in our study, in other words, organic grape juice had higher concentrations of total polyphenols, catechin and resveratrol [15]. In our study, the polyphenols chlorogenic acid, taxifolin, ferrulic acid, naringin, rutin, quercetin, luteolin, kaempferol, apigenin and epicatechin were not identified in either extract. However, Dani *et al.* (2010) [8] detected the presence of the polyphenols rutin, naringin, quercetin and kaempferol as well. This can be explained by differences in extraction methods used in the current and previous studies [8]. This was also evident in a study conducted by Orhan *et al.* (2007) [31] with leaves of *Vitis vinifera*, which found that the amounts of phenolic compounds at end of the process depended on the solvent used in the extraction. For example, extraction with chloroform yielded lower amounts of total polyphenols compared to extraction with ethanol [31].

**Table 1:** Content of total polyphenols, catechin and resveratrol in *V. labrusca* leaf extracts using organic and conventional grapevines.

EXTRACT	Total Polyphenols (mg/100 g)	Catechin (mg/100 g)	Resveratrol (mg/100 g)
CONVENTIONAL	19.83 ± 0.76	211.82 ± 5.05	0.01 ± 0.003
ORGANIC	81.79 ± 2.68*	161.10 ± 0.97 *	0.04 ± 0.004 *

* Statistical difference, *p* < 0.05.

**Figure 1:**
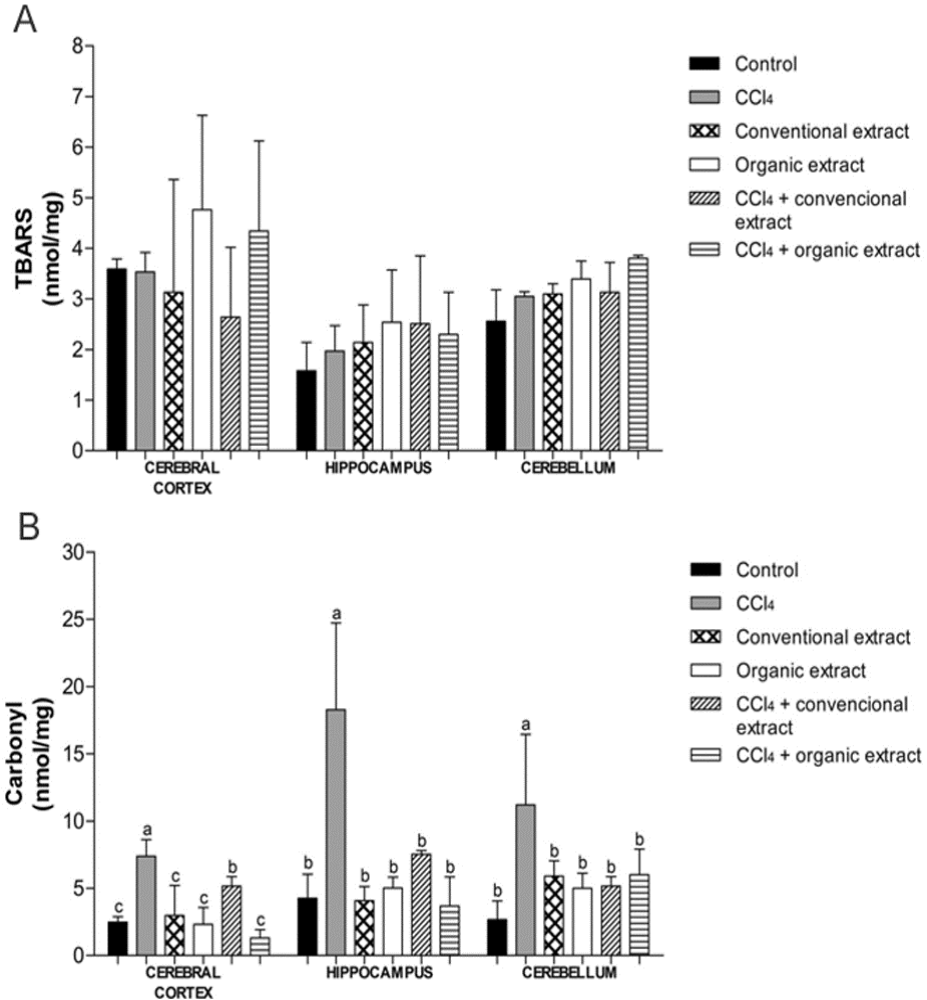
(**A**) Levels of thiobarbituric acid reactive species (TBARS) (nmol/mg) and (**B**) Carbonyl (nmol/mg protein) in brain tissues of rats treated with different extracts (organic and conventional) and carbon tetrachloride (3 mL/kg). Data are expressed as mean ± SD. Different letters indicate statistical difference according to ANOVA and Tukey’s post-test (*p* < 0.05) for each tissue evaluated.

In the last years, these compounds have been the subject of much research because they generate many benefits to human health [32]. The benefits of these bioactive compounds include reducing the risk of liver, cardiovascular and neurodegenerative diseases, as well as exerting antioxidant and anticarcinogenic actions [33]. Along this line, the human brain is often in contact with reactive molecules produced by the normal metabolism of oxygen or derived from exogenous sources, which are known as reactive oxygen species [34]. Thus, the central nervous system has been considered to be predisposed to oxidative damage [35], mainly by possessing a high concentration of lipids and requiring a high consumption of oxygen, so it is a potential damage target for oxidative stress [9,35,36]. To assess the biological activity of these extracts, we evaluated their neuroprotective effect through their ability to inhibit lipid peroxidation and protein oxidation as well as by modulation of antioxidant enzyme activity. In our study, there was no statistical difference between the treatments evaluated with regard to lipid peroxidation, demonstrating that CCl_4_ was not able to induce damage to lipids in any of the tissues evaluated (Figure 1A). However, in another study, lipid damage induced by CCl_4_ in the striatum and substantia nigra brain of Wistar rats was reduced with the administration of organic and conventional grape juice [24]. Lipid peroxidation was also reduced in an *in vivo* experiment performed with *Vitis vinifera* grapevine leaf extract in rat liver, where levels of lipid peroxidation decreased significantly after treatment of animals with the extract in question [31]. The same was demonstrated in *in vitro* studies conducted with grapevine leaf extracts (*Vitis labrusca*), conventional and organic. It was observed that levels of lipid peroxidation damage generated by the damage inducer also decreased after treatment with the extracts in the cerebral cortex, hippocampus, cerebellum, kidney, heart and liver [7,8].

The protein carbonyl assay showed that CCl_4_ increased protein damage in all tissues, and both extracts were able to reduce these levels. In the hippocampus and cerebellum, both extracts reduced damage to proteins in the same proportion, though in the cerebral cortex, the organic extract produced a more significant reduction in protein oxidation as compared to conventional extract (Figure 1B). Our data corroborated the results of another study conducted with similar *V. labrusca* leaf extract but that study was conducted with tissue *in vitro* [7]. In a study carried out with grape juice, protein oxidation was also decreased in the group receiving the juice [24].

**Figure 2:**
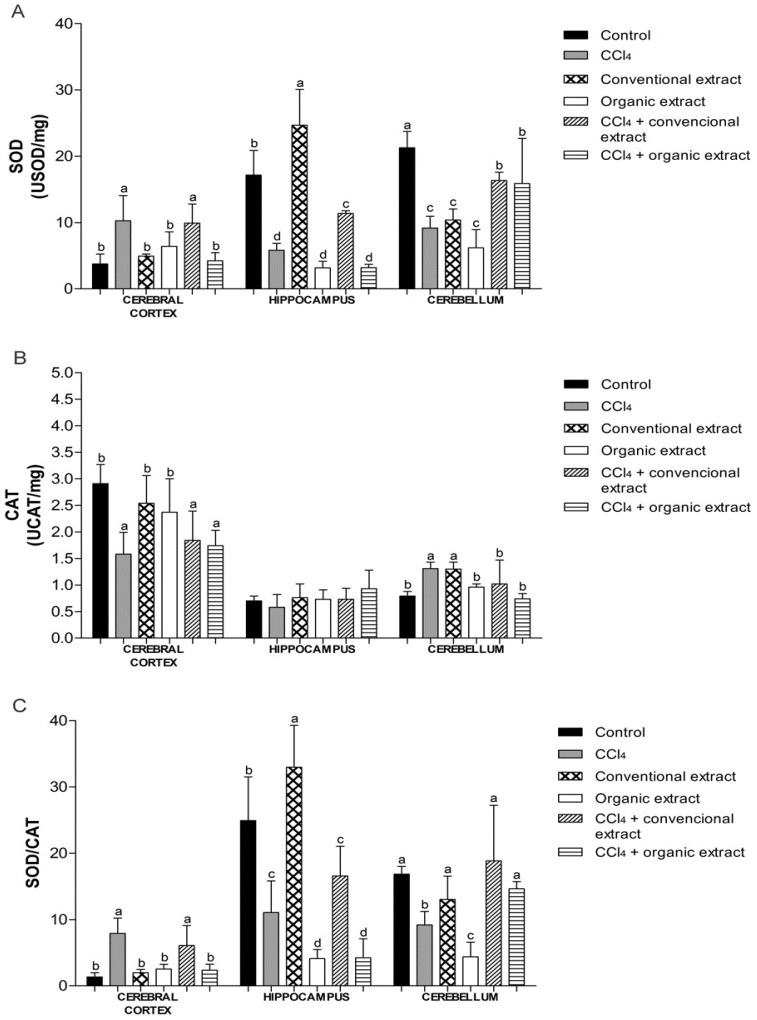
(**A**) Superoxide dismutase (SOD) activity (USOD/mg protein) (**B**) Catalase (CAT) activity (UCAT/mg protein) and (**C**) SOD/CAT ratio in brain tissues of rats treated with different extracts (organic and conventional) and carbon tetrachloride (3 mL/kg). Data are expressed as mean ± SD. Different letters indicate statistical difference according to ANOVA and Tukey’s post-test (*p* < 0.05) for each tissue evaluated.

Cells possess antioxidant defenses, including the enzymes SOD and CAT [11]. We observed that the treatment with CCl_4_ increased SOD activity in cerebral cortex, while reducing activity in other tissues (Figure 2A). In the cerebral cortex, only the organic extract was able to restore enzyme activity altered by CCl_4_, to similar levels as the control group. The organic extract was also shown to be effective against damage caused by CCl_4_ in the hippocampus. This decrease caused by the organic extract was not observed in the cerebellum. In this tissue, the damage induced did not cause an increase in SOD activity. Nevertheless, in the cerebellum, the conventional extract was able to increase SOD activity, and when only extracts were administered, both reduced the activity of this enzyme, as did CCl_4_. However, in the groups that received the extracts and damage inducer, there was an increase in SOD level, but it remained below the control value. Another study showed that organic grapevine leaf extract increased SOD activity in kidney and liver and increased CAT activity in heart [7]. Increased SOD levels have also been observed in rat plasma with an extract of *Vitis coignetiae Pulliat* [37] and in rat liver and kidney with an extract of *Vitis vinifera* [11].

With regard to CAT activity, we observed that the CCl_4_ reduced enzyme activity in the cerebral cortex, which was not reversed by treatment with extracts (Figure 2B).

Similarly, in another study, grapevine leaf extract also reduced CAT activity in cerebral cortex exposed to H_2_O_2_ *in vitro* [8]. The groups treated only with extracts behaved similarly to the control group. In the cerebellum, CCl_4_ caused an increase in CAT activity, while enzyme activity levels in the other groups were the same as the control. In the hippocampus, no difference was observed between the treatments. Conventional and organic grape juice was found to inhibit the decrease in SOD and CAT activity induced by pentylenetetrazol, a potent convulsant drug [15].

Besides SOD and CAT activities, we also evaluated the relationship between these enzymes. The SOD/CAT ratio is an essential parameter to check if there is an imbalance between these two enzymes (Figure 2C). An imbalance between antioxidant defenses may generate increased oxidative stress and thus cause various diseases [38]. In the cerebellum, both extracts restored SOD/CAT, whereas in the cerebral cortex, only the organic extract maintained this ratio at a similar level as the control group. This finding could be attributed to the higher polyphenol content of the organic extract. Still, in the cerebral cortex, we observed an increase in this ratio in the CCl_4_ group, as well as with the inducing agent combined with conventional extract. We also noted that this ratio in this tissue in the other experimental groups was lower, which was also observed in a study of the effect of grape juice on the striatum and substantia nigra of rats [24]. This behavior was not observed in the hippocampus. This difference in antioxidant enzyme activity, lipid damage and oxidative protein damage, according to the brain structure evaluated, as previously mentioned in other studies [39,40]. This difference can be attributed, for example, to the differences in blood supply in each of the different brain regions [41]. However, further studies are needed for better understanding of the sensitivity of each of the structures evaluated.

## 4. Conclusions

Thus, the results of this study suggest that the treatment of rats with grapevine leaf extract could be effective against protein oxidative damage and in reestablishing the SOD/CAT ratio in central nervous system tissues, which when excessive or unbalanced, respectively, can contribute to neurodegenerative disorders.
